# Occludin Promotes Adhesion of CD8^+^ T Cells and Melanocytes in Vitiligo via the HIF-1*α* Signaling Pathway

**DOI:** 10.1155/2022/6732972

**Published:** 2022-02-16

**Authors:** Puyu Zou, Yangfan Xiao, Qiancheng Deng, Yaqian Shi, Ruixuan You, Zixin Pi, Jiani Liu, Yi Zhan, Qinghai Zeng, Zhuotong Zeng, Rong Xiao

**Affiliations:** ^1^Department of Dermatology, Second Xiangya Hospital, Central South University, Changsha, Hunan Key Laboratory of Medical Epigenetics, China; ^2^Department of Anesthesiology, Second Xiangya Hospital, Central South University, Changsha, China; ^3^Clinical Nursing Teaching and Research Section, Second Xiangya Hospital, Central South University, Changsha, China; ^4^Department of Dermatology, The First Affiliated Hospital, Zhejiang University School of Medicine, Hangzhou, China; ^5^Department of Dermatology, Third Xiangya Hospital, Central South University, Changsha, China

## Abstract

Loss of melanocytes induced by activated CD8^+^ T cells is the pathological hallmark of vitiligo. Melanocyte-specific CD8^+^ T cells are recruited to the skin via chemokines, thereby releasing perforin, granzyme, and other cytotoxic substances that destroy the melanocytes. However, the mechanism of CD8^+^ T cells to adhere to melanocytes is unknown. Previous transcriptome sequencing results published by our group showed that the occluding (*OCLN)* gene was significantly upregulated in CD8^+^ T cells from skin lesions of vitiligo. Occludin is a crucial component of the tight junction between cells; in cells without tight junction, occludin mediates the adhesion of two cells in the form of a self-ligand. This study demonstrated that *OCLN* gene expression was elevated in the CD8^+^ T cells of vitiligo patients, and occludin mediates the adherence of CD8^+^ T cells to melanocytes. Besides, pathological changes in vitiligo skin lesions reveal that CD8^+^ T cells continuously persist in the skin lesions, which is related to the persistence of the disease. In this regard, we found that fibroblasts from vitiligo patients significantly express occludin, which may participate in the continuous retention of CD8^+^ T cells in the skin lesions. The pathogenesis of vitiligo is closely related to oxidative stress, and our data suggest that overexpression of hypoxia-inducible factor-1*α* (HIF-1*α*) increases the expression of occludin. Besides, ChIP-qPCR of CD8^+^ T cells revealed that HIF-1*α* directly binds to the *OCLN* promoter. Thus, occludin upregulation promotes the adhesion of CD8^+^ T cells and melanocytes via the HIF-1*α* signaling pathway. Our study results suggested a critical role for *OCLN* in the occurrence, progression, and maintenance of vitiligo. Therefore, inhibiting the expression of *OCLN* gene may be a potential targeted treatment strategy.

## 1. Introduction

Vitiligo is characterized by skin depigmentation and selective destruction of melanocytes [1, 2]. Complex interactions of genetic predisposition, environmental triggers, biochemical factors, oxidative stress, and immunologic factors have been implicated in vitiligo pathogenesis [3–5]. Activated CD8^+^ T cells play a key role in the occurrence and progression of the disease [6–8]. Melanocyte-specific CD8^+^ T cells are recruited to the skin via chemokines, thereby releasing perforin, granzyme, and other cytotoxic substances that destroy the melanocytes [6–10]. However, the mechanism of CD8^+^ T cells to adhere to melanocytes is unknown.

In our previous study, we directly extracted CD8^+^ T cells from the skin lesions of vitiligo patients and normal people and performed transcriptome sequencing and filtering for differentially expressed genes in these lymphocytes. The sequencing results showed that the *OCLN* gene was significantly upregulated in the CD8^+^ T cells from vitiligo skin lesions [11].

Occludin (OCLN) is a tetraspan redox-sensitive protein associated with tight junctions [12]. It plays an important role in lymphocyte migration and adhesion. Occludin is hardly expressed in normal lymphocytes. However, stimulation with phorbol 12-myristate 13-acetate (protein kinase C activator) and A23187 (a combination of calcium ionophores) increases the expression of occludin in activated lymphocytes (CD4^+^ T and CD8^+^ T cells) [13]. Besides, Saito et al. found that increased occludin expression in activated *γδ* T cells was involved in the migration of *γδ* T cells from the epidermis to the lymph nodes [14]. Another study reported that *γδ* intraepithelial lymphocytes bind to the occludin on the intestinal epithelial cells through the occludin on its surface, so as to migrate rapidly and extensively in the intraepithelial compartment [15]. Occludin mediates cell adhesion through a unique self-ligand mechanism (see supplementary materials figure S1). Interestingly, OCLN was upregulated in melanocyte precursors of vitiligo patients via transcriptome sequencing [16]. However, it remains unclear whether occludin, a membrane protein, mediates the adhesion of lymphocytes and melanocytes, thereby assisting CD8^+^ T cells to kill melanocytes.

Oxidative stress plays an important role in the pathogenesis of vitiligo. And the expression of hypoxia-inducible factor (HIF-1*α*) also increases in CD8^+^ T cells from patients with vitiligo. However, the relationship between HIF-1*α* and OCLN remains to be further explored.

In this study, we examined the expression level of OCLN in CD8^+^ T cells from vitiligo patients and normal controls. We further explored whether occludin mediates the adhesion of CD8^+^ T lymphocytes and melanocytes. We also investigated the regulatory mechanism of OCLN expression. This will provide new insights into the pathogenesis of vitiligo.

## 2. Materials and Methods

### 2.1. Subjects

Peripheral blood and skin biopsy samples from nonsegmental vitiligo patients were collected from the Department of Dermatology in Second Xiangya Hospital of Central South University, China. Assessment of disease activity was done according to the vitiligo disease activity (VIDA) score. The stages of these enrolled vitiligo patients are active or rapid progressive. Patients who had received systemic therapy for more than three months or topical medication for more than a month were excluded from the study. We also recruited an equal number of age- and sex-matched controls. The unaffected control skin was collected using plastic surgery. Peripheral blood samples were also collected from healthy volunteers. We obtained informed consent from all patients. The study protocols were approved by the ethics committee.

### 2.2. CD8^+^ T Cell Culture and Transfection

CD8^+^ T lymphocytes were separated from PBMCs by CD8^+^ T MicroBeads (Miltenyi Biotec, Germany). Then, the isolated CD8^+^ T lymphocytes were stimulated with plate-bound anti-CD3 and anti-CD28 Abs and cultured in complete RPMI 1640 medium (Servicebio, China) for 48 h. Meanwhile, every 1 × 10^7^ normal control CD8^+^ T lymphocytes were treated with 1 *μ*g OCLN plasmid using a Human T Cell Nucleofector Kit and an Amaxa nucleofector (Lonza) for 48 h (GenScript, Shanghai, China) [17]. Every 5 × 10^6^ vitiligo CD8^+^ T cells were transfected with 50 nM siRNA using riboFECT™ CP for 48 h (RiboBio, China).

### 2.3. Multispectrum Immunohistochemistry

Samples were fixed in formalin and embedded in paraffin. The antigen was retrieved using citrate buffer and heat-induced retrieval method. The sections were incubated with anti-human CD8 (1 : 500; Abcam, Cambridge, MA, USA), anti-human occludin (1 : 82; Invitrogen, Carlsbad, CA, USA), and anti-human Melan-A (1 : 1000; Abcam) primary antibodies overnight at 4°C. The slides were stained for 15 min with secondary antibodies and fluorophore using the Opal 7 color IHC detection kit (PerkinElmer, Hopkinton, MA, USA). The slides were counterstained with DAPI for 10 min (1 : 2000; Abcam). Skin slides were scanned using PerkinElmer Vectra, and images were analyzed using the inForm software (PerkinElmer).

### 2.4. Western Blot

Specimens were lysed in a mixture of RIPA buffer and 1% cocktail (CWBIO, Beijing, China). Protein concentrations were determined using a BCA Protein Assay Kit (Thermo Fisher Scientific, Waltham, MA, USA). Proteins were separated by 4–20% SDS-PAGE and transferred to polyvinylidene fluoride, followed by incubation with 5% nonfat milk for 1 h. The membranes were incubated with anti-human OCLN (1 : 1000 dilution; Invitrogen, 404700), anti-human HIF-1*α* (1 : 1000; Cell Signaling Technology (CST), Berkeley, CA, USA; D2U3T), Melan-A (1 : 1000, Abcam, ab210546), flag (1 : 1000; CST; D6W5B), and anti-GAPDH (1 : 5000 dilution; Abcam; ab9485) overnight at 4°C. Horseradish peroxidase-conjugated anti-rabbit IgG antibody (1 : 5000 dilution; CST; 7074) served as a secondary antibody. Band intensity was detected using the Syngene G:BOX Chemi XX6 (Syngene, Cambridge, UK).

### 2.5. Real-Time Quantitative PCR (qPCR)

The extraction and reverse transcription of total RNA were performed as previously described [11]. Real-time quantitative PCR was performed using the SuperReal PreMix Plus (TIANGEN, Beijing, China; FP205). The relative expression level of perforin was normalized to a *β*-actin internal control and analyzed using the 2^−ΔΔ*Ct*^ method.

### 2.6. Epidermal Melanocyte Culture

Foreskin samples were cleaned of subcutaneous fat and cut into several small pieces. Each sample was digested using 2 mg/mL dispase II solution (Sigma-Aldrich, St. Louis, MO, USA). The samples were submerged epidermis-side-down overnight at 4°C. The epidermis and dermis were separated, and the epidermis was soaked in 5 mL of 0.25% trypsin and EDTA solution (Gibco, Grand Island, NY, USA) for 10 min. RPMI 1640 medium (Gibco, USA) was used to neutralize the trypsin solution. Cells obtained from the filtration with a 40 mm screen mesh were cultured in M254 medium (Gibco, USA).

### 2.7. Adhesion Assays

Melanocytes were seeded on growth cover glasses (Corning Inc., Corning, NY, USA). Confluent melanocyte monolayers were treated with M254 media for 24 h. CD8^+^ T lymphocytes with different treatments were added to melanocyte monolayers and allowed to adhere for 12 h at 37°C. Nonadherent cells were washed away. Adherent cells were fixed by 4% formalin solution and lysed with 0.5% Triton X-100. After being blocked by BSA for 30 min, the growth cover glasses were probed with anti-human OCLN antibody (1 : 82 dilution; Invitrogen; 404700), anti-human CD8 (1 : 1000; Abcam), and anti-human Melan-A (1 : 1000; Abcam) for 1 h. The secondary antibody staining was performed using an Opal 7 color IHC detection kit (PerkinElmer). DAPI was used to visualize the cell nuclei. Skin slides were scanned using the PerkinElmer Vectra, and images were analyzed using the inForm software (PerkinElmer) [18–20].

### 2.8. Flow Cytometry and Apoptosis Analysis

Apoptosis assay was conducted in melanocytes after mixing culture with CD8^+^ T cells to detect dead cells. They were stained with annexin V and PI (4A Biotech, Beijing, China) and detected by BD FACSCanto. The data were analyzed using the FlowJo software (Tree Star Inc., San Carlos, CA, USA).

### 2.9. Skin Explant

Healthy skin samples were cut into 4 mm^2^ fragments, and the subcutaneous fat was removed by scissors. Fragments were placed in 24-well plates, with the epidermis facing upwards. Skin explant fragments were placed at Dulbecco's modified Eagle's medium (DMEM) (Sigma-Aldrich) containing 1 × 10^6^ CD8^+^ T cells with different treatments and antibiotics at 37°C with 5% of carbonic gas for 24 h. The fragments were fixed in 4% formalin solution and used to create paraffin blocks. All other steps were similar to those performed for multispectrum immunohistochemistry [21–23].

### 2.10. Reactive Oxygen Species (ROS) Detection

Intracellular ROS were detected using DCFH-DA (Reactive Oxygen Species Assay Kit, Beyotime, S0033S). 100 *μ*M of DCFH-DA was added to CD8^+^ T cells under hypoxia and incubated in the dark for 30 min at 37°C. The cells were washed twice with phosphate-buffered saline (PBS). Fluorescence was measured using a fluorescence microscope with an excitation wavelength of 488 nm and an emission wavelength of 525 nm.

### 2.11. ChIP-qPCR

ChIP-qPCR was performed using the Simple ChIP kit (CST). Each IP sample consisted of 4 × 10^6^ cells. DNA and proteins were crosslinked. Then, chromatin was sheared into 200–500 bp fragments. Anti-H3 (CST), normal human IgG (CST), and anti-human HIF-1*α* (CST) were added to each IP sample. DNA was recovered and purified using a chelating resin. Real-time PCR was used to identify and quantify the immunoprecipitated sequences.

### 2.12. Statistical Analysis

All statistical analyses were performed using the GraphPad Prism 6.0 (GraphPad Inc., San Diego, CA, USA). Two-tailed unpaired Student's *t*-test was used to compare the difference between the two groups. Data are presented as mean ± standard error of the mean (SEM). *P* < 0.05 was considered to be statistically significant.

## 3. Results

### 3.1. OCLN Is Highly Expressed in CD8^+^ T Cells and Melanocytes of Vitiligo Patients

**Figure 1:**
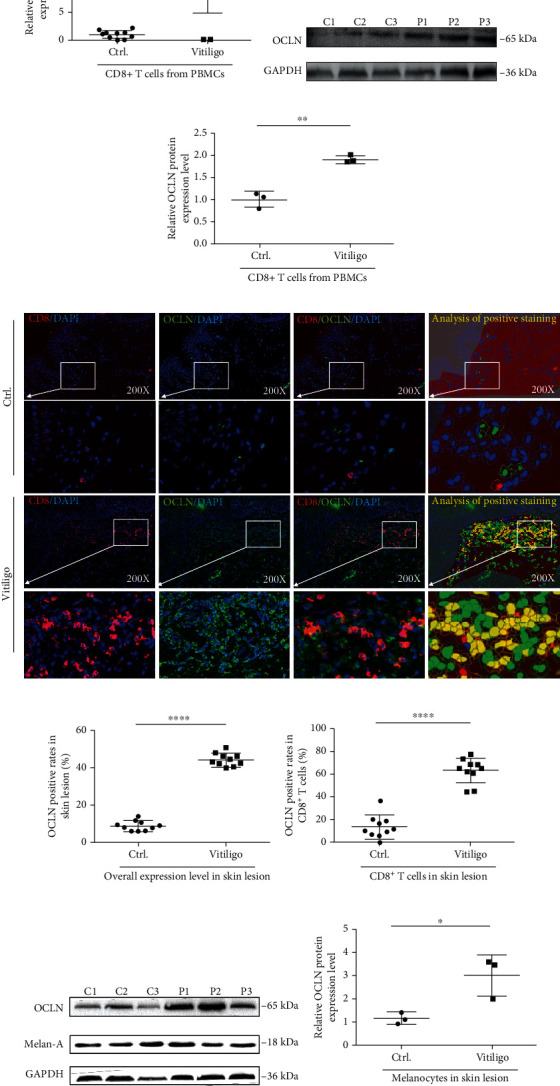
(a) The mRNA expression level of OCLN in CD8^+^ T cells in the peripheral blood of normal people and patients with vitiligo (*n* = 10, ^∗∗∗^*P* < 0.001). (b, c) The protein expression level of OCLN in CD8^+^ T cells in the peripheral blood of normal people and patients with vitiligo (*n* = 3, ^∗∗^*P* < 0.01, C: control; P: patient). (d–f) The expression of CD8 and OCLN in normal skin and skin lesions of patients with vitiligo. (*n* = 10, ^∗∗∗∗^*P* < 0.0001, ^∗∗∗∗^*P* < 0.0001). The red mark represents CD8-positive staining, the green mark represents OCLN-positive staining, and the blue mark represents DAPI. The yellow mark of image analysis of positive staining represents the part where CD8 and OCLN are colocalized after analysis by Inform2.3 software. (g, h) The expression of OCLN in melanocytes from normal skin and skin lesions of patients with vitiligo (*n* = 3, ^∗^*P* < 0.05, C: control; P: patient).

Previous sequencing results reported by our group showed that OCLN expression was upregulated in vitiligo patients [11]. Real-time PCR and Western blotting were used to verify the consistency of OCLN mRNA and protein expression levels in CD8^+^ T cells from vitiligo patients' peripheral blood mononuclear cells (PBMCs). It was found that OCLN was highly expressed in the CD8^+^ T cells of vitiligo patients' PBMCs (Figures 1(a)–1(c)).

In addition, CD8 and OCLN markers were used to double stain the vitiligo skin lesions and normal skin tissues using multispectral immunohistochemistry. Compared to normal controls, the OCLN expression level was significantly upregulated in the CD8^+^ T cells from vitiligo skin lesions (Figures 1(d)–1(f)). Meanwhile, occludin was upregulated in melanocytes located in perilesions of vitiligo patients compared to healthy controls (Figures 1(g) and 1(h)).

### 3.2. Occludin Expression Promotes CD8^+^ T Cell Adhesion to Melanocytes in Mixed Culture

**Figure 2:**
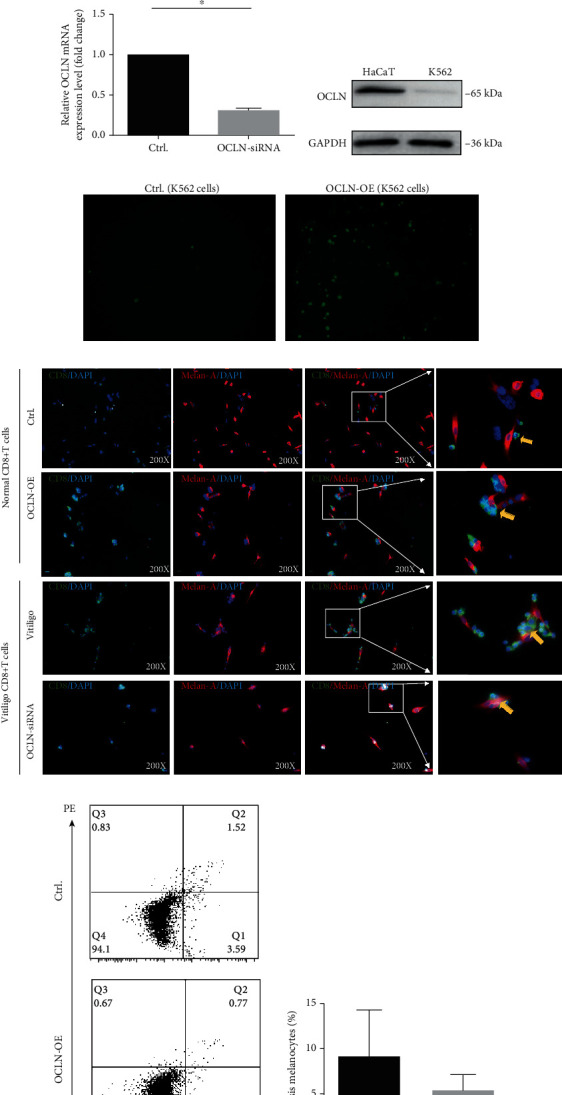
(a, b) The protein expression level of OCLN in normal control group and CD8^+^ T cells transfected with OCLN overexpression plasmid (*n* = 3, ^∗∗∗∗^*P* < 0.0001, C: control; O: OCLN-OE). (c) The mRNA expression level of OCLN in vitiligo CD8^+^ T cells and vitiligo CD8^+^ T cells transfected with OCLN siRNA (*n* = 3, ^∗^*P* < 0.05). (d) The expression level of OCLN in normal K562 cells and HaCaT cells. (e) The adhesion rate of melanocytes and normal K562 cells or K562 cells transfected with OCLN overexpression plasmid. (f) The adhesion rate of CD8^+^ T cells to melanocytes (*n* = 3, ^∗^*P* < 0.05, ^∗^*P* < 0.05, ^∗^*P* < 0.05). The red mark represents Melan-A-positive staining, the green mark represents CD8-positive staining, and the blue mark represents DAPI. (g, h) The apoptosis of melanocytes after coculture with CD8^+^ T cells (*n* = 3, *P* > 0.05).

OCLN was successfully overexpressed in CD8^+^ T cells by plasmid transfection, and OCLN expression was successfully interfered by siRNA transfection (Figures 2(a)–2(c)). The adhesion effect of OCLN was first confirmed in K562 cells, a type of cell line that does not express OCLN (Figure 2(d)). After mixing culture of normal K562 cells and melanocytes, we found that K562 cells do not significantly adhere to melanocytes. However, after mixing culture of melanocytes and K562 cells transfected with OCLN plasmid, the adhesion ratio significantly increased compared to normal K562 cells (Figure 2(e)). To investigate the role of OCLN in CD8^+^ T cells, the adhesion of melanocytes to the CD8^+^ T cells was examined. The results showed that compared to normal CD8^+^ T cells (adhesion ratio: 27.42 ± 7.73%), a higher ratio of vitiligo CD8^+^ T cells adhered to melanocytes (adhesion ratio: 377.14 ± 87.81%). After CD8^+^ T cells were transfected with OCLN plasmid in the mixed culture system, the adhesion ratio of CD8^+^ T cells significantly increased compared to normal CD8^+^ T cells (adhesion ratio: 182.21 ± 29.48%). Remarkably, melanocytes exhibited no difference in their apoptosis rate after being cocultured with CD8^+^ T cells treated with OCLN plasmid or normal CD8^+^ T cells. In contrast, CD8^+^ T cells exhibited a significant decline in adhesion to melanocytes after transfection with OCLN siRNA (adhesion ratio: 37.62 ± 5.51%; Figures 2(f)–2(h)).

### 3.3. Increased Infiltration of CD8^+^ T Cells in Skin Explants Cocultured with CD8^+^ T Cells Overexpressing OCLN

**Figure 3:**
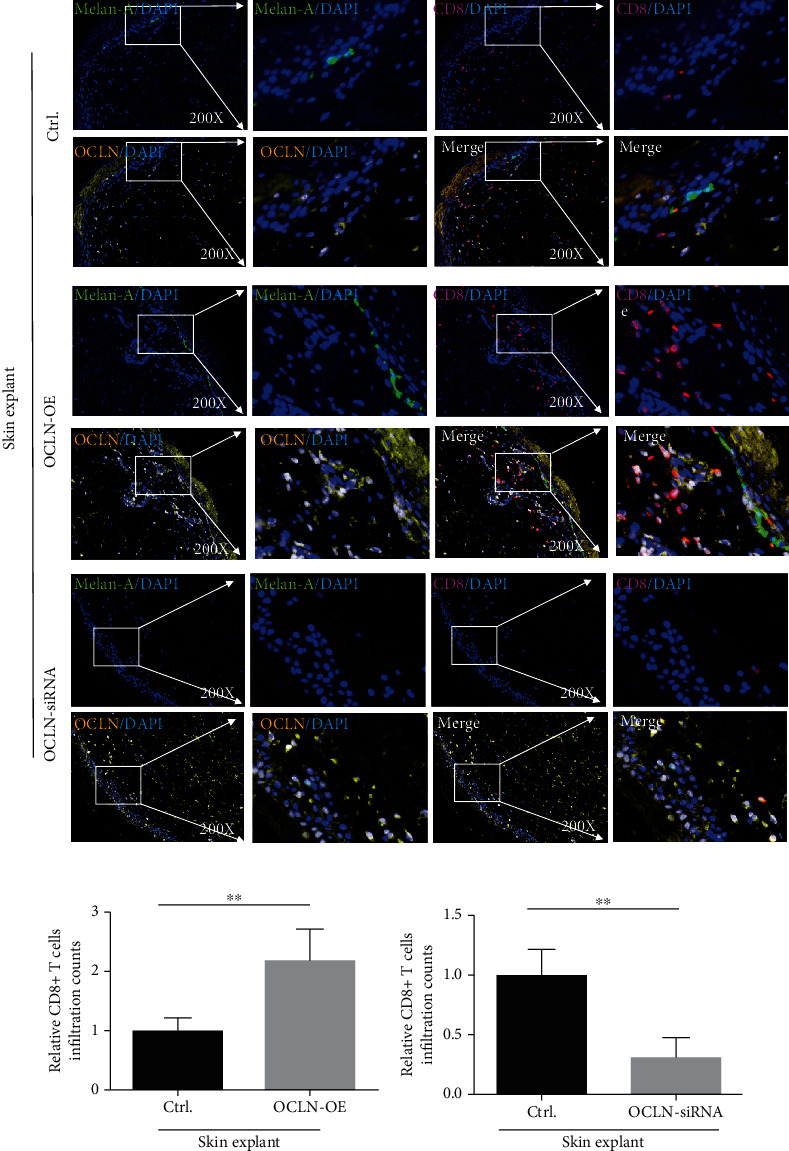
The skin infiltration of CD8^+^ T cells in skin explants (*n* = 4, ^∗∗^*P* < 0.01, ^∗∗^*P* < 0.01). The red mark represents CD8-positive staining, the green mark represents Melan-A-positive staining (a melanocyte-specific antibody), the blue mark represents DAPI, and the yellow mark and white mark represent OCLN-positive staining, because when yellow light and blue light overlap, it is displayed as white.

To further investigate whether occludin mediates the adhesion of CD8^+^ T cells to melanocytes, normal skin explants were cultured in a medium containing CD8^+^ T cells overexpressed with occludin or CD8^+^ T cells interfered with OCLN siRNA, and immunofluorescence assay was performed on the paraffin sections of the skin explants. Compared to normal controls, cocultured CD8^+^ T cells overexpressing OCLN and skin explants demonstrated increased infiltration of CD8^+^ T cells in skin explants. In contrast, it showed a decreased infiltration of CD8^+^ T cells in skin explants when cocultured skin explant with CD8^+^ T cells interfered with OCLN siRNA (Figure 3).

### 3.4. Fibroblasts Enriched with Occludin Maintain CD8^+^ T Cells in the Skin Lesion

**Figure 4:**
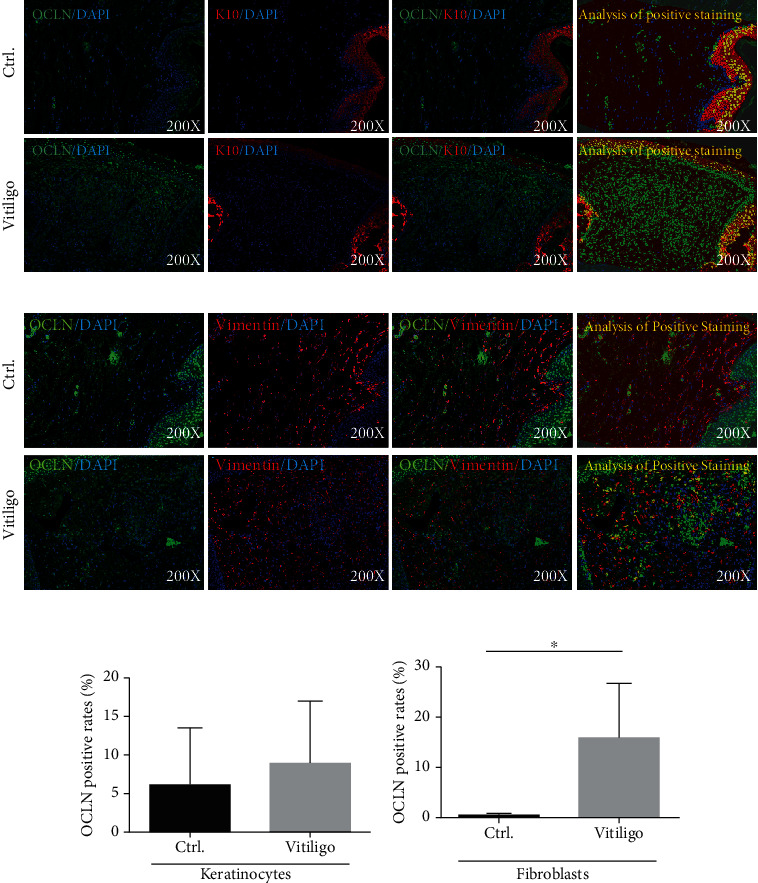
(a, b) The protein expression level of OCLN in keratinocytes from vitiligo patients and normal control (*n* = 5, *P* > 0.05). The red mark represents K10-positive staining, the green mark represents OCLN-positive staining, and the blue mark represents DAPI. The yellow mark of images analysis of positive staining represents the part where K10 and OCLN are colocalized after analysis by Inform2.3 software. (c, d) The protein expression level of OCLN in fibroblasts from vitiligo patients and normal control (*n* = 5, ^∗^*P* < 0.05). The red mark represents vimentin-positive staining, the green mark represents OCLN-positive staining, and the blue mark represents DAPI. The yellow mark of image analysis of positive staining represents the part where vimentin and OCLN are colocalized after analysis by Inform2.3 software.

In previous immunofluorescence assays, it was found that occludin was also expressed in non-CD8^+^ T cells. To determine the type of cells expressing occludin, as well as their effects on the migration and retention of CD8^+^ T cells, multispectral immunohistochemistry was used to stain skin lesion with vimentin (a fibroblast marker), K10 (a keratinocyte marker), and occludin. Occludin was significantly upregulated in the fibroblasts from vitiligo patients compared to healthy controls. However, no difference was found in the OCLN expression of keratinocytes. Keratinocytes from both vitiligo patients and normal controls had highly expressed occludin (Figure 4).

### 3.5. Occludin Upregulation Promotes the Adhesion of CD8^+^ T Cells to Melanocytes via the HIF-1*α* Signaling Pathway

**Figure 5:**
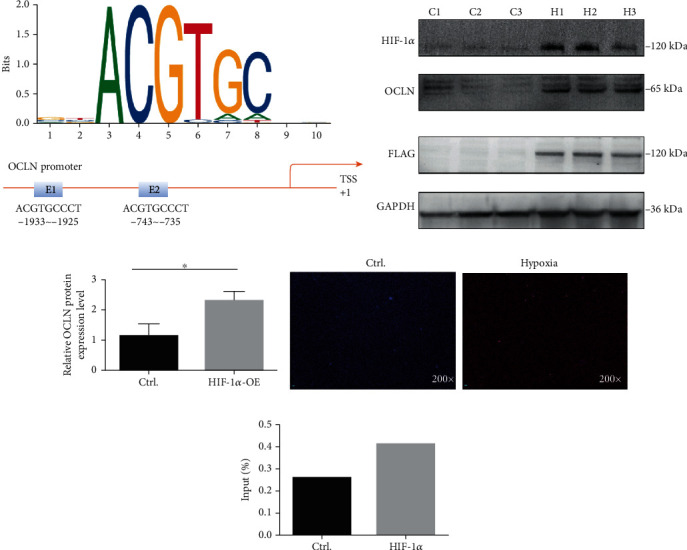
(a) HIF-1*α* sequence and binding sites (E1 and E2) to the OCLN promoter region were predicted by using JASPAR database (http://jaspar.genereg.net/). (b, c) The protein expression levels of HIF-1*α*, OCLN, and FLAG in CD8^+^ T cells overexpressed with HIF-1*α* plasmid and normal control (*n* = 3, ^∗^*P* < 0.05, C: control; H: HIF-1*α*-OE). (d) ROS level of CD8^+^ T cells under normoxia and hypoxia. (e) HIF-1*α* ChIP-qPCR analyses of CD8^+^ T cells, fold change = 1.76.

The sequence 1500 bp upstream of the 5′ untranslated region sequences (UTRs) of OCLN was analyzed using JASPAR database (http://jaspar.genereg.net/), and the results showed a large number of binding sites of the conserved transcription factor hypoxia-inducible factor-1*α* (HIF-1*α*) (Figure 5(a)). Interestingly, our preliminary study found that HIF-1*α* was high ranked in vitiligo CD8^+^ T cells [10].

After overexpressing HIF-1*α* in normal CD8^+^ T cells from PBMCs, Western blotting was used to detect the occludin expression. It was found that HIF-1*α* overexpression in CD8^+^ T cells elevated OCLN protein expression (Figures 5(b) and 5(c)). To further explore whether HIF-1*α* directly binds to the OCLN promoter, ChIP-qPCR was performed in CD8^+^ T cells under hypoxia. We could find that under hypoxic conditions, ROS levels are significantly increased (Figure 5(d)). In chromatin fractions pulled down by anti-HIF-1*α* antibody, only one site of the OCLN promoter was detected. The sequence for this site was GGACGTGCCT (Figure 5(e)).

## 4. Discussion

Vitiligo is an acquired disease characterized by skin depigmentation, which affects about 0.5%–2% of the general population [24, 25]. Patients with vitiligo often suffer from heavy psychological burden because the skin lesions occur in the exposed areas [26, 27].

In vitiligo, CD8^+^ T cells are necessary and sufficient to kill melanocytes. Melanocyte-specific CD8^+^ T cells migrate to the skin under the action of chemokines and destroy melanocytes by releasing perforin and granzymes [8, 28, 29]. However, after CD8^+^ T cells migrate to the skin under the action of chemokines, how it further adheres to melanocytes and kills melanocytes remains unknown.

Previously, we performed transcriptome sequencing of CD8^+^ T cells and found upregulation of OCLN in CD8^+^ T cells in vitiligo skin lesions [11]. In this study, we confirmed that the expression level of OCLN was significantly increased in CD8^+^ T cells of vitiligo patients by qPCR, Western blotting, and multispectral immunohistochemistry. This suggests that OCLN might be involved in the pathogenesis of vitiligo.

As mentioned before, increased occludin expression in activated *γδ* T cells enhancing the migration of *γδ* T cells from the epidermis to the lymph nodes, thus, upregulated occludin in CD8^+^ T cells from peripheral blood indicating that it may facilitate CD8^+^ T cells from blood migrating to the skin. Previous studies have shown that occludin plays an important role in the adhesion of lymphocytes to epithelial cells, as well as of exogenous occludin to Xenopus embryo cells in the form of self-ligands [12, 30, 31]. Therefore, we assumed that CD8^+^ T cells from PBMCs of vitiligo patients migrate to the skin, where occludin on the surface of CD8^+^ T cells may bind to the occludin on the surface of melanocytes to mediate the adhesion of CD8^+^ T cells and melanocytes. In this study, we tested this assumption using adhesion assays. The adhesion ratio of mixed cultured melanocytes with CD8^+^ T cells overexpressing OCLN was significantly higher than that of controls.

Skin explant experiments were performed to further verify the adhesion theory. Skin explants were placed in a medium containing CD8^+^ T cells overexpressing OCLN, which showed increased CD8^+^ T cell infiltration into the skin lesion. These results showed that OCLN expression on CD8^+^ T cells enhanced the adhesion of CD8^+^ T cells to skin melanocytes. OCLN narrowed the physical distance between CD8^+^ T cells and melanocytes, which may contribute to melanocyte destruction.

In the aforementioned multispectral immunohistochemistry assays, CD8^+^ T cells expressing OCLN and non-CD8^+^ T cells expressing OCLN were significantly increased in the skin lesions of vitiligo patients. However, melanocytes in the skin lesions of vitiligo patients had been destroyed. Moreover, occludin mediates cell adhesion in the form of self-ligands. Therefore, after melanocytes are being massively destroyed, other cells may participate in the continuous retention of CD8^+^ T cells in the skin lesions by overexpressing occludin. Multispectral immunohistochemistry confirmed that OCLN expression significantly increased in the fibroblasts of vitiligo patients. This indicated that after a large number of melanocytes are destroyed, fibroblasts may maintain CD8^+^ T cells in the skin lesions through occludin on the surface of their cell membranes. The recent paper published by Xu et al. also showed that fibroblasts play an important role in the pathogenesis of vitiligo, which further testifies to this article's point of view [32].

In vitiligo, decolorized leukoplakia often returns at the same location after treatment is discontinued, and tissue-resident memory T cell (T_rm_) cell, a subtype of CD8^+^ T cell that resides in the skin, plays a key role in this process [33]. As mentioned above, OCLN plays an important role in the continuous retention of CD8^+^ T cells in the skin lesions. OCLN may also participate in maintaining the residence of T_rm_ in the vitiligo skin lesions, which explains why vitiligo always recurs at the same location. The role of OCLN in T_rm_ cells remains to be further explored. In summary, OCLN plays an important role in the occurrence and maintenance of vitiligo, and OCLN may be a potential treatment target of vitiligo.

We used a website (http://jaspar.genereg.net/) to explore the reason for the high OCLN expression in vitiligo CD8^+^ T cells and found a large number of binding sites of HIF-1*α* in the OCLN promoter region. HIF-1*α* is a transcription factor that is sensitive to oxygen [34]. Additionally, OCLN is a redox-sensitive protein [35]. Interestingly, our group previously found increased HIF-1*α* expression in the peripheral CD8^+^ T cells and vitiligo skin lesions [10]. Therefore, HIF-1*α* is probably a transcription factor that regulates OCLN expression. In this study, we confirmed that HIF-1*α* overexpression in normal CD8^+^ T cells increased OCLN expression. Next, we used ChIP-qPCR to demonstrate that the transcription factor HIF-1*α* and OCLN share the binding site. HIF-1*α* binds to the OCLN promoter, thereby confirming the ability of HIF-1*α* to regulate OCLN induction. Thus, occludin was upregulated in vitiligo via the HIF-1*α* signaling pathway.

Several recent studies have shown that oxidative stress plays a key role in the pathogenesis of vitiligo. In addition to directly causing melanocyte apoptosis, it can also induce autoimmune responses that destroy melanocytes, which initiates vitiligo [4, 36, 37]. Excessive accumulation of reactive oxygen species plays an important role in the pathogenesis of vitiligo oxidative stress. Meanwhile, HIF-1*α* acts as the core of the “cellular oxygen sensor system,” and hypoxia conditions induce its massive expression. However, the increase in reactive oxygen species inhibits HIF-1*α* degradation and increases the nuclear transfer of this transcription factor, so that HIF-1*α* is enhanced in both quantity and function [38–40]. Thus, oxidative stress in vitiligo drives increased HIF-1*α* expression in CD8^+^ T cells, and HIF-1*α* overexpression and enhancement upregulate occludin expression, which mediates the adhesion of CD8^+^ T cells to melanocytes. HIF-1*α* was also highly expressed in fibroblasts under the hypoxic environment, which may explain the increased OCLN expression in the fibroblasts from vitiligo patients [41]. These data identify a critical role of OCLN in vitiligo occurrence, progression, and maintenance. Therefore, OCLN inhibition may become a targeted treatment strategy.

## 5. Conclusion

In this manuscript, we demonstrated that abnormally highly expressed occludin mediates the adhesion of CD8^+^ T lymphocytes and melanocytes. Fibroblasts highly expressing occludin may participate in the continuous retention of CD8^+^ T cells in the skin lesions. Oxidative stress in vitiligo drives increased HIF-1*α* expression, and HIF-1*α* overexpression and enhancement upregulate occludin expression, which mediates the adhesion of CD8^+^ T cells to melanocytes. Our study results suggested a critical role for OCLN in the occurrence, progression, and maintenance of vitiligo. Therefore, OCLN inhibition may become a targeted treatment strategy.

## Data Availability

The datasets presented in this study can be found in online repositories. The names of the repository/repositories and accession number(s) can be found in the article.

## Supplementary Materials

Supplementary Materials S1 Line drawing of the junctional complex (left): occludin exhibits a multidomain tetraspan structure and interacts with the ZO-1 GUK domain. The function of occludin in tight junction and nontight junction (right): in tight junction, occludin contributes to barrier function, and in nontight junction, occludin mediates cell adhesion.
