# Supplementary material for: Occludin Promotes Adhesion of CD8+ T Cells and Melanocytes in Vitiligo via the HIF-1α Signaling Pathway
Source: Oxid Med Cell Longev. 2022 Feb 16;2022:6732972. doi: 10.1155/2022/6732972 (PMC8865978; doi:10.1155/2022/6732972)
Supplement: Supplementary Materials — S1 Line drawing of the junctional complex (left): occludin exhibits a multidomain tetraspan structure and interacts with the ZO-1 GUK domain. The function of occludin in tight junction and nontight junction (right): in tight junction, occludin contributes to barrier function, and in nontight junction, occludin mediates cell adhesion. [file 6732972.f1.pptx]

## Slide 1
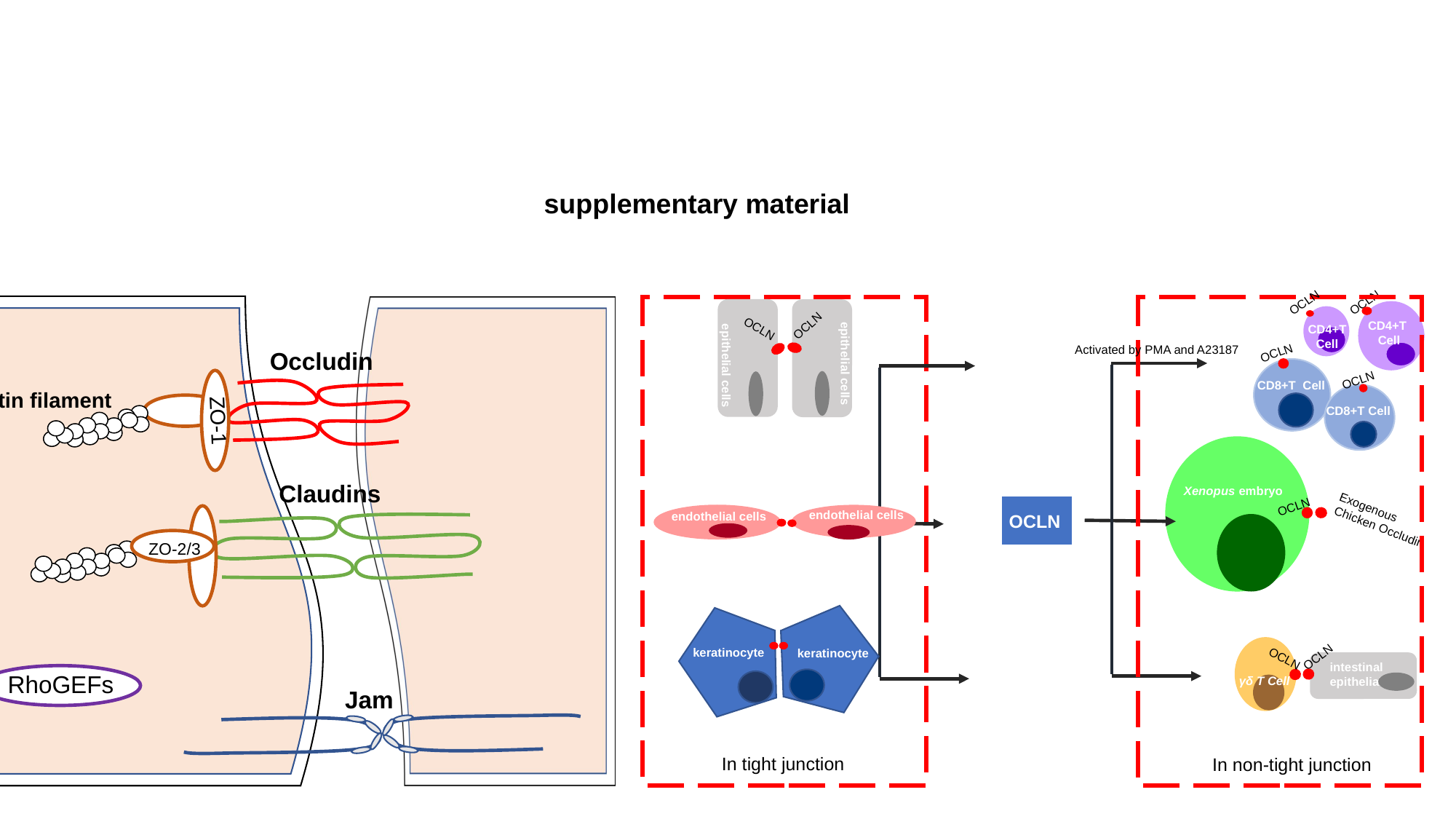

supplementary material
OCLN
CD4+T Cell
OCLN
CD4+T
Cell
OCLN
 epithelial cells
 epithelial cells
OCLN
Activated by PMA and A23187
OCLN
CD8+T Cell
OCLN
CD8+T Cell
Xenopus embryo
OCLN
OCLN
Exogenous
Chicken Occludin
endothelial cells
endothelial cells
γδ T Cell
keratinocyte
keratinocyte
OCLN
intestinal epithelial cells
OCLN
In tight junction
In non-tight junction
Occludin
ZO-1
Actin filament
Claudins
ZO-2/3
RhoGEFs
Jam
